# Coverage optimization of soil moisture wireless sensor networks based on adaptive Cauchy variant butterfly optimization algorithm

**DOI:** 10.1038/s41598-022-15689-3

**Published:** 2022-07-08

**Authors:** Jinyan Liang, Min Tian, Yang Liu, Jie Zhou

**Affiliations:** 1 College of Mechanical and Electrical Engineering, Shihezi University, Shihezi, 832000 China; 2 College of Information Science and Technology, Shihezi University, Shihezi, 832000 China

**Keywords:** Information technology, Computer science, Electrical and electronic engineering

## Abstract

Soil moisture wireless sensor networks (SMWSNs) are used in the field of information monitoring for precision farm irrigation, which monitors the soil moisture content and changes during crop growth and development through sensor nodes at the end. The control terminal adjusts the irrigation water volume according to the transmitted information, which is significant for increasing the crop yield. One of the main challenges of SMWSNs in practical applications is to maximize the coverage area under certain conditions of monitoring area and to minimize the number of nodes used. Therefore, a new adaptive Cauchy variant butterfly optimization algorithm (ACBOA) has been designed to effectively improve the network coverage. More importantly, new Cauchy variants and adaptive factors for improving the global and local search ability of ACBOA, respectively, are designed. In addition, a new coverage optimization model for SMWSNs that integrates node coverage and network quality of service is developed. Subsequently, the proposed algorithm is compared with other swarm intelligence algorithms, namely, butterfly optimization algorithm (BOA), artificial bee colony algorithm (ABC), fruit fly optimization algorithm (FOA), and particle swarm optimization algorithm (PSO), under the conditions of a certain initial population size and number of iterations for the fairness and objectivity of simulation experiments. The simulation results show that the coverage rate of SMWSNs after ACBOA optimization increases by 9.09%, 13.78%, 2.57%, and 11.11% over BOA, ABC, FOA, and PSO optimization, respectively.

## Introduction

Nowadays, with the development of precision agriculture, the use of wireless sensor networks to better perform the task of sensing and acquiring information about the farmland environment is an important research topic for scholars^1^. The basis for implementing precision farm irrigation is the collection and processing of crop soil environment information. SMWSNs, due to their low node cost, flexible structure, self-organizing networks, and wireless technology communication can accurately acquire crop soil information and transmit it effectively, then the control terminal precisely adjusts the irrigation water volume based on the collected data^2^. Coverage, as a fundamental issue in wireless sensor networks, mainly reflects the quality of sensing that the sensor network can provide. Optimizing the coverage of SMWSNs is important for the rational allocation of network space resources to better accomplish information acquisition and accurate irrigation^3^.

Traditional farm management uses uniform irrigation times and volumes, whereas the crop growing environment in a farm is constantly changing over time and space. In response to this uncertainty, precision agriculture, based on modern information technology, can accurately access various parameters in the agricultural environment at any time and anywhere, which directly affect the growth of crops^4^. The information monitored by the SMWSNs is processed and aggregated by the precision agriculture management system, enabling timely detection of fluctuations in soil moisture content and rapid and precise control of irrigation water^5^. More importantly, the application not only improves water utilization but also greatly increases crop yields and promotes economic benefits. Therefore, SMWSN is increasingly used in the field of precision crop irrigation management.

SMWSNs are widely used in agricultural irrigation management, but when nodes are improperly deployed can lead to the problem of untimely information collection and transmission^6^. Optimizing the node distribution in wireless sensor networks through effective node deployment algorithms to improve the coverage rate of the network is one of the important elements in wireless sensor network research. For large-scale agricultural fields, most of them utilize the random deployment of sensor nodes by UAVs, which saves a lot of human and material resources. However, randomly deployed sensor nodes are often unevenly distributed, resulting in coverage voids or coverage redundancy in some areas, which seriously affects data transmission and causes huge energy loss^7^. Researchers use mobile sensor node coverage optimization to alleviate the above problems and achieve full coverage of the monitoring area as far as possible, where typical research algorithms mainly include virtual force algorithm, computational geometry algorithm and intelligent optimization algorithm^8^.

This approach is effective in reducing monitoring blind spots and wasting node resources by optimizing the deployment of node locations with algorithms for a given number of nodes. The above problems can be improved by deploying dynamic nodes or scheduling algorithms to adjust node locations, which can improve network coverage performance and reliability. Compared with the traditional analytical and iterative methods, the swarm intelligence algorithm is less parametric and easier to implement and has good global optimization capabilities^9^. However, traditional swarm intelligence algorithms have some shortcomings, for example, the existing genetic algorithm (GA)^10^, simulated annealing algorithm (SA)^11^, and particle swarm (PSO)^12^ have poor accuracy, tend to fall into local optimum, and slow convergence rate when optimizing the network coverage. The above algorithms need to be further improved and applied to the coverage optimization of SMWSNs to achieve nearly complete coverage.

A main objective of this paper is to maximize the coverage rate of the target detection area by using a certain number of sensor nodes and optimizing the locations where they will be deployed, i.e., to find the optimal locations of these nodes so that the improved algorithm is applicable to the coverage of SMWSNs and the field of agricultural irrigation management. In this context, a new coverage optimization model for SMWSNs is developed and a new adaptive Cauchy variational butterfly optimization algorithm (ACBOA) is designed to optimize the network. Specifically, this paper effectively improves the BOA algorithm for its shortcomings and applies it to the coverage optimization problem by adaptively adjusting the deployment of sensor nodes in wireless sensor networks to obtain more uniform distribution and higher coverage in the monitoring area. A series of simulation experiments demonstrate the effectiveness of the algorithm in improving the coverage rate of nodes.

The main contributions of this paper are as follows.

1. A new coverage optimization model for SMWSNs that integrates sensor coverage and network quality of service is developed. The model innovatively combines coverage rate, different number of nodes and sensor node sensing range for SMWSNs coverage optimization. In addition, the model realistically and objectively reflects the coverage rate of the monitored area and the effectiveness of agricultural irrigation control management.
2. A new optimization factor based on the effective coverage rate of SMWSNs is used to construct an objective function is devised. The objective function innovatively takes network coverage as the evaluation index and abstracts the complex coverage problem into a high-dimensional vector-seeking optimal solution problem. This initiative greatly reduces the difficulty of the coverage problem and accelerates the running time of the algorithm.
3. A major innovative adaptive Cauchy variant butterfly optimization algorithm (ACBOA) is applied to SMWSNs coverage optimization. New Cauchy variants and adaptive factors for improving the global and local search ability of ACBOA, respectively, are designed. More importantly, ACBOA achieves the approximate minimum number of deployed nodes for coverage optimization of SMWSNs in the same monitoring area, which innovation not only saves the deployment cost of nodes, but also dramatically reduces the energy consumption of nodes.

The rest of the paper is structured as follows: “Related work” presents related research on the SMWSNs coverage optimization problem. “System model” shows the SMWSNs coverage optimization model. “ACBOA for improved coverage rate of SMWSNs” proposes ACBOA to solve the SMWSNs coverage optimization problem. “Results and discussion” demonstrates the effectiveness of ACBOA in improving coverage rate through simulation experiments and discusses the results. Conclusions are given in “Conclusions”.

## Related work

Node deployment is one of the key technologies in wireless sensor networks, and the use of sensor technology to solve practical application problems relies heavily on the information that can be collected at the sensor's location. However, the lack of computing and communication capabilities of SMWSNs nodes and the uncontrolled deployment of some nodes can cause many interruptions to information collection. The most important design metric in optimizing node deployment is therefore efficiency, which can be achieved by ensuring coverage rate while reducing the number of working SMWSNs nodes to provide the collected data^13–15^. The traditional approach to sensor node deployment is to deploy static nodes on a large scale, and too many static nodes can lead to conflict in communication. In this case, mobile nodes can be used to improve the situation^16^. Typical research algorithms for mobile node coverage currently include virtual force algorithms (VFA)^17^, computational geometry algorithms^18^, and intelligent optimization algorithms^19^.

The concept of virtual force is applied to the dynamic analysis of nodes by Zou et al.^20^ to enhance the coverage of sensor nodes after random deployment. The process of VFA for network coverage optimization is that the node moves according to its forces until the forces are balanced or until it reaches the boundary of the monitored area. The movement of sensor nodes is controlled by the distance between them. When the distance between nodes is less than a certain distance threshold they will repel each other and move the nodes in the opposite direction, when the distance between nodes is greater than a threshold they will attract each other and move the nodes in that direction. Thus, the nodes are evenly distributed in the monitored area driven by the combined forces of attraction and repulsion, and the node coverage area is effectively expanded. However, Zou et al. did not consider the problem of sensor nodes out of bounds and the researchers proposed to increase the repulsive force of the boundary on the sensors to further ensure that the nodes are effectively deployed in the monitoring area.

Computational geometry algorithms exploit the geometric relationships between sensor nodes to optimize network coverage blindness, two of the most commonly used algorithms are Voronoi diagrams and Delaunay triangles. Voronoi diagrams are widely used for node coverage optimization due to their ability to reduce computational dimensionality, and Delaunay triangles are dual diagrams of Voronoi diagrams and are unique. Ghahroudi et al.^21^ proposed a Voronoi-based collaborative node deployment (VCOND) algorithm that provides maximum coverage while ignoring the initial position of the nodes. The algorithm addresses the dependency of the initial deployment of sensor nodes by defining two criteria, neighborhood density and sensor coverage rate, which means that these two criteria reflect the relationship between the areas in the vicinity of each sensor node, and using this relationship allows VCOND to take into account the node neighborhood situation when terminating iterations. The VCOND-based algorithm solves the initial deployment dependency well and improves the final coverage rate, but there are still local coverage gaps between sensors resulting in low network uniformity. Therefore, Tao et al.^22^ proposed to use of Delaunay triangular networks to construct hollow circles for detecting coverage holes between sensors and relocating them, which further improves the network coverage by repairing the coverage holes with VFA. In this research context as the demand for information in harsh environments grows, traditional stochastic deployment approaches are no longer suitable for SMWSNs coverage optimization. Mainstream virtual force algorithms have demonstrated unique advantages in mobile node deployment, but the primary objective of node coverage today is to deploy a small number of nodes to maximize the coverage of the network.

Intelligent optimization algorithms are global and heuristic algorithms that are widely used in node coverage problems because of their global optimization, generality, and applicability to parallel processing. More and more researchers are applying biomimetic intelligent population algorithms to SMWSNs coverage optimization, such as genetic algorithms, particle swarm algorithms, ant colony algorithms, and so on. These algorithms are of great interest to researchers because of their ability to solve many complex problems with high optimization and stability. The particle swarm algorithm (PSO) was proposed by Kennedy et al.^23^ in 1995 and is simpler in structure and design than the genetic algorithm. For these reasons the Particle Swarm algorithm is easier to obtain an approximate optimal solution than the Genetic Algorithm and is also easier to implement and more efficient to run. Improvements to the particle swarm algorithm mainly address the inherent drawbacks of being prone to premature maturity and falling into local optimality. Kong et al.^24^ proposed a wireless sensor network coverage improvement method based on the enhanced PSO algorithm by designing dynamic adjustment of inertia coefficients and variational operators to improve the traditional PSO algorithm, increasing the global convergence speed and global diversity and avoiding it from falling into local optimality. Wang et al.^25^ proposed a novel mobile sensor-based node deployment algorithm that combines coverage hole repair and PSO to improve the coverage rate of wireless sensor networks and reduce network energy consumption. The algorithm first randomly deploys sensor nodes in the monitoring area and then keeps the nodes stationary or switches to sleep mode, secondly divides the network into grids and calculates the coverage rate of each grid and selects the network with lower coverage rate as a candidate grid, and finally wakes up the mobile sensors from sleep mode to fix the coverage holes and calculates the mobile location of the nodes using PSO. Nguyen Thi Hanh et al.^26^ improved the individual representation, heuristic initialization and local search to improve the area coverage rate based on the genetic algorithm, and secondly used area integral as a fitness function to improve the simulation reliability. Coverage and lifetime are the two most important issues in wireless sensor networks. Idrees et al.^27–33^ proposed various algorithms to increase the network lifetime while ensuring network coverage and complete data monitoring for as long as possible, which is of value and significance for practical applications in agricultural irrigation. The above findings show that although the results of applying various population intelligence algorithms to node coverage optimization have improved, further improvements are needed to achieve near-complete coverage before applying them to SMWSNs coverage optimization.

Existing research has shown the value and significance of using novel and improved intelligent optimization algorithms for node deployment problems^34^. The butterfly optimization algorithm is a novel population intelligence algorithm proposed by Arora et al.^35^ and has been applied to wireless sensor network node localization problems^36^, optimization training of wavelet neural networks^37^, etc. In this paper, based on the original butterfly algorithm, a combination of smaller adaptive operators for improving the butterfly's local search capability and the Cauchy variant for improving the algorithm's global search capability and increasing the ability of the search space, coverage optimization of SMWSNs achieves the purpose of working with the minimum number of nodes while ensuring maximum coverage.

## System model

### SMWSNs overlay mathematical model

1. Cover method

**Figure 1:**
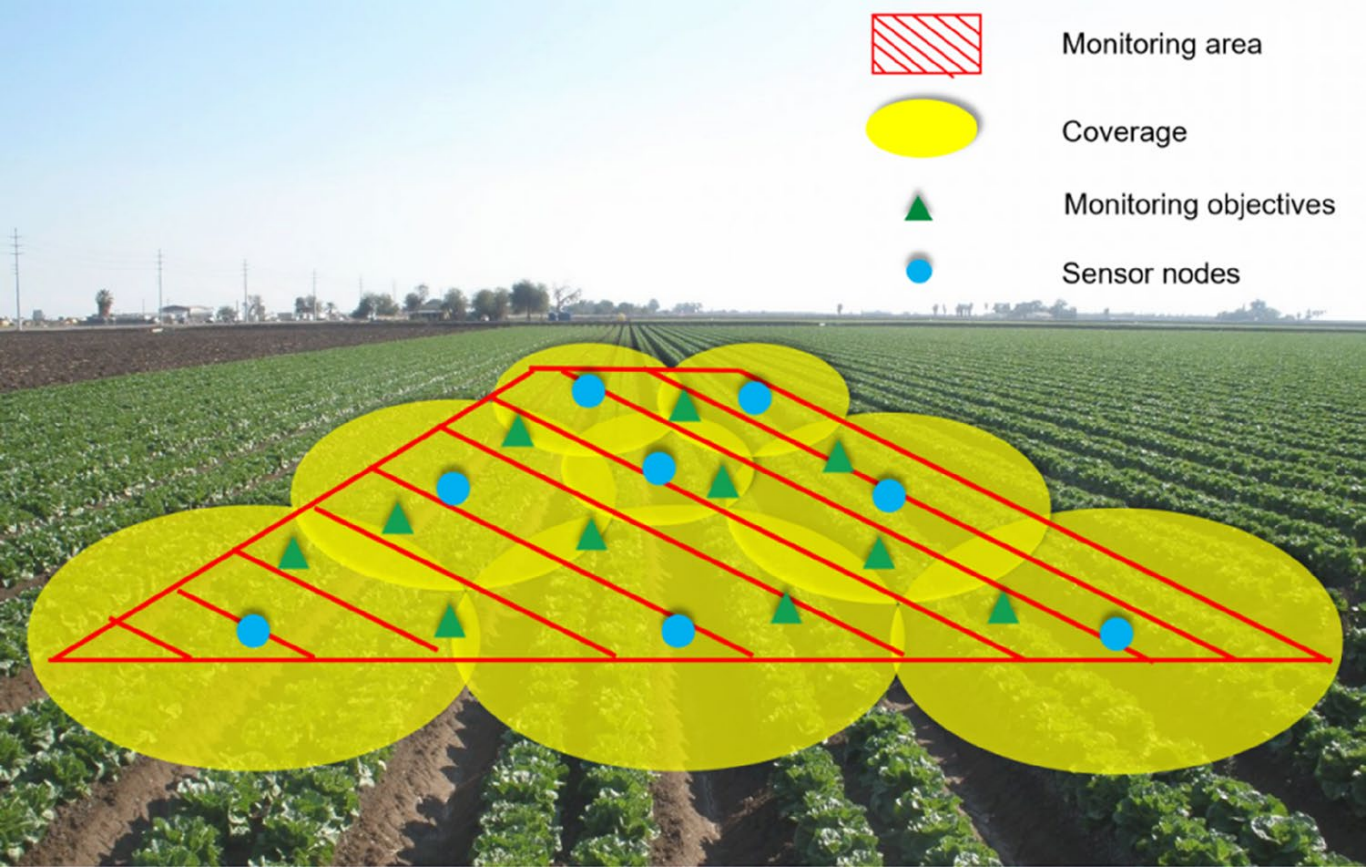
Regional coverage model.

Depending on the application environment and coverage method of SMWSNs, the coverage methods can be classified as point coverage, fence coverage, and area coverage. Specifically, point coverage is for some discrete target points, fence coverage is for the moving trajectory of moving targets, and area coverage is for the overall coverage of a monitoring area. Based on the characteristics of SMWSNs, this paper chooses area coverage for monitoring the soil moisture environment in farming areas. This coverage control aims to maximize the coverage of a limited number of sensor nodes, enabling the sensor network to monitor data for the entire sub-region within the target range at all times. The area coverage model is shown in Fig. 1.

(2) Mathematical model

The main objective of SMWSNs coverage rate optimization is to deploy an approximate minimum number of nodes to improve coverage and thus reasonably allocate network space resources to better fulfill the task of environmental sensing and information acquisition. The main idea of SMWSNs in practical application is to ensure the accuracy and reliability of information by sending and receiving information through several information aggregation nodes and wireless sensor nodes scattered in the monitoring area, and the agricultural irrigation management system analyses the received data to obtain valid information.

The monitoring area is assumed to be a two-dimensional plane, and sensor nodes can be deployed anywhere within the area. To facilitate implementation this paper describes the simplified mathematical model as:

1. The sensor nodes are randomly deployed in the monitoring area with negligible mass and volume.
2. The sensor nodes apply a Boolean perception model.
3. The sensing radius of the sensor nodes is $R$.

Selecting the swarm intelligence algorithm to optimize the location of the nodes randomly thrown in the monitoring area can accomplish effective coverage of the entire monitoring area. The sensor node deployment problem can be described as knowing the locations of all nodes and the number of sensor nodes to be deployed and adjusting the node locations to maximize the coverage rate.

### Sensor node sensing model

Agricultural irrigation management systems use the rate of data transmission between nodes as an important factor in the selection of a sensing model. The Boolean sensing model can control the sensing results between nodes through Boolean expressions, which improves the efficiency of sensing between nodes. Assume that the monitoring area Z of SMWSNs is a two-dimensional plane and the monitoring area Z is a square area of m*m. N isomorphic sensors are deployed in this region, i.e. the nodes all have the same parameter information and all have the same sensing radius R and communication radius ${R}_{c}$. To ensure the connectivity of the SMWSNs, the parameter relationship of the nodes is ${R}_{c}=2R$. the set of nodes can be expressed as $C=\{{c}_{1},{c}_{2},\cdots ,{c}_{N}\}$, where ${c}_{i}=({x}_{i},{y}_{i})$ denotes the position of the node coordinates as $\{{x}_{i},{y}_{i}\}$ and the position of the sensing target $T=(x,y)$. then the distance between the sensing node and the sensing target is shown in Eq. (1).

1 $$d\left( {c_{i} ,T} \right) = \, \sqrt {\left( {x_{i} - x} \right)^{2} + \left( {y_{i} - y} \right)^{2} }$$

**Figure 2:**
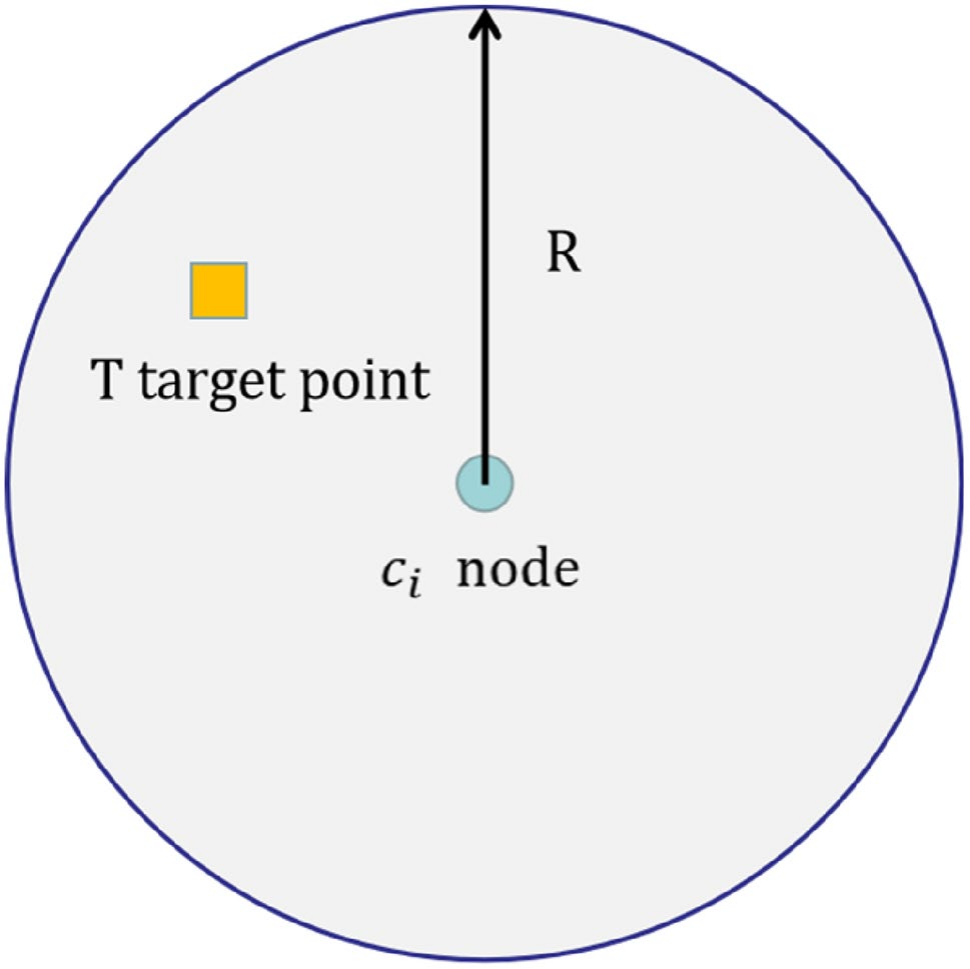
Boolean perception model.

In this paper, the Boolean model is used as the node perception model, as shown in Fig. 2, i.e. the coverage rate of the sensing node ${c}_{i}$ for the target point $(x,y)$ is only 0, 1. The probability of perception of node ${c}_{i}$ for $T$ is denoted by ${p}_{cov}({c}_{i},T)$. When the position of $T$ is within the perception range of node ${c}_{i}$, the perception probability is 1; otherwise, the perception probability is 0. Then the probabilistic model of pixel coverage by sensor nodes is shown in Eq. (2).

2 $$p_{{\text{cov}}} \left( {c_{i} , \, T} \right) = \, \left\{ \begin{gathered} 1, \, \quad if \, d\left( {c_{i} , \, T} \right) < R \hfill \\ 0,\quad otherwise \hfill \\ \end{gathered} \right.$$

### Probabilistic evaluation indicators

SMWSNs coverage control techniques are used to enhance the reliability of monitoring data across the network while reducing node energy consumption. However, coverage control techniques can also have negative effects on the network, such as increasing the complexity of node operations and generating additional energy consumption, so metrics are needed to measure the effectiveness of coverage control techniques. The distribution of sensor nodes, the number of nodes, and the amount of energy consumed by the nodes all affect coverage performance, so there is a need to investigate the appropriate distribution of nodes and a smaller number of nodes to optimize the coverage of the SMWSNs.

### Coverage rate

Usually, the sensor perception probability of a target is less than 1. To increase the perceived probability of a target, multiple sensors are required to detect it collaboratively. The joint sensing probability of all sensor nodes for a given target is then shown in Eq. (3).

3 $$p_{cov} \left( {C, \, T} \right) \, = \, 1 - \, \prod\limits_{{c_{i} \in C}} {\left( {1 - \, p_{cov} \left( {c_{i} , \, T} \right)} \right)}$$

where $\mathrm{C}$ denotes all wireless sensor nodes in the monitoring area. Area coverage rate is one of the main evaluation metrics of the coverage model and is expressed as the ratio of the coverage area of the node-set to the total area of the monitoring area, and the area coverage rate of the node-set is noted as ${R}_{cov}(C)$.

4 $${\text{R}}_{cov} \left( C \right) \, = \, \frac{{\sum P_{cov} (C)}}{LW}$$

where ${P}_{cov}(C)$ is the sum of target point probabilities for all sensor nodes covered. Monitoring area assuming the rectangular area is $LW {\mathrm{m}}^{2}$.

### Uniformity

Uniformity is also a measure of network coverage quality, reflecting the uniformity of node distribution. Uniformity is calculated as shown in Eqs. (5) and (6).

5 $$U = \frac{1}{n}\sum\limits_{i = 1}^{n} {U_{i} }$$

6 $$U_{i} = \left( {\frac{1}{{k_{i} }}\sum\limits_{i = 1}^{{k_{i} }} {\left( {D_{i,j} - M_{i} } \right)^{2} } } \right) \, ^{{1/2}}$$

where $n$ is the total number of nodes, and ${k}_{i}$ is the number of neighbor nodes of node $i$. ${D}_{i,j}$ is the Euclidean distance between nodes $i,j$; ${M}_{i}$ is the average distance between node $i$ and all its neighbors. The smaller the network coverage uniformity, the more average the energy consumption and the better the coverage rate quality.

### Coverage efficiency

Coverage efficiency is used to measure the utilization of sensor nodes and reflects both the redundancy of the network and the energy consumed by the network as a whole. It is defined as the proportion of the sum of the coverage of all nodes in the monitoring area to the sum of the coverage of multiple nodes. Calculate the coverage efficiency as shown in Eq. (7).

7 $$r_{cov} = \, \frac{{ \cup P_{cov} \left( C \right)}}{{\sum P_{cov} \left( C \right)}} \,$$

The relationship between coverage efficiency and coverage ratio is shown in Eq. (8).

8 $$r_{{\text{cov}}} = \, \frac{{R_{{\text{cov}}} \left( C \right) \times \, LM}}{{N \, \times \, \pi R^{2} }}$$

### Connectivity

The ability to communicate effectively between nodes in SMWSNs network is a prerequisite for the integrity of the overall monitoring network. Due to the self-organizing nature of wireless sensor networks and the fact that each node has to inter-communicate the collected information with each other. Network connectivity plays a very important role in the reliability of monitoring data. Excellent connectivity is the basis for the effectiveness of coverage optimization algorithms.

### Covering problem function

From the above, the description of the requested SMWSNs coverage rate mathematical model problem is shown in Eq. (9).

9 $$f\left( I \right) \, = \, Max\left( {R_{cov} \left( I \right)} \right)$$

This function indicates that the solution to the coverage problem of SMWSNs is to find the maximum coverage of this mathematical model. Coverage optimization focuses on the node deployment strategy to collect data information for the monitoring range, using the coverage rate magnitude to represent the degree of coverage of the nodes over the target range. Specifically, the study of the SMWSNs coverage problem is generally approached from two perspectives: firstly, to determine the effective coverage of the target area by nodes to collect and transmit data information in the target area, and secondly, to increase the network coverage rate with a reasonable allocation of space resources to ensure high utilization of node resources. The nodes are deployed by the distribution of monitoring points in the target area so that the nodes can monitor the target area with maximum coverage rate, and at the same time ensure that the nodes consume less while the coverage rate is high, thus allowing the nodes to collect and transmit data for a long time.

## ACBOA for improved coverage rate of SMWSNs

To optimize the node coverage rate, an adaptive Cauchy variational butterfly optimization algorithm (ACBOA) is presented in this paper. The butterfly optimization algorithm (BOA) is simple to operate and has few parameters to adjust^35,38^, it also suffers from the problems of easily falling into local optimum and poor convergence performance. To make BOA suitable for solving SMWSNs coverage optimization problems, this paper uses a variation of the global position update of the butterfly using the Cauchy distribution function to improve the global search ability of the algorithm and introduces an adaptive inertia weight factor at the local update to improve the local mining ability of the algorithm. Improving on the two problems of traditional BOA's tendency to fall into local optimum and convergence performance, this paper uses ACBOA for SMWSNs coverage optimization to achieve a smaller number of working nodes, thus reducing network consumption.

The process of ACBOA includes initializing the population, calculating the fitness value, calculating the butterfly individual scent concentration, global and local position updating, the Cauchy variation operation, and adding adaptive weights operation.

### Initializing the population

The first step in the pre-swarm intelligence algorithm is to initialize the population. The ACBOA-based SMWSNs coverage optimization focuses on how to deploy nodes to maximize network coverage rate with a known number of sensor nodes, i.e., to find the optimal location of known nodes in the target area and approximate to achieve full coverage of the target area. With dim sensors deployed in the region, the set of nodes can be expressed as

10 $$X=({X}_{1},{X}_{2},...,{X}_{dim})$$

The expression for randomly generating individual initial solutions in N-dimensional search space is shown in Eq. (11).

11 $${X}_{i}=u+(u-l)\cdot z$$

where ${X}_{i}$ denotes the spatial location of the $i$th butterfly in the group, $u$ denotes the upper bound of the search space, $l$ denotes the lower bound of the search space, and $z$ denotes a matrix of random numbers between $(0,1)$. In ACBOA, each individual has its own unique sensory and individual perception capabilities, and they move randomly or towards the individual that emits the most scents. In this way, the butterflies share information and form a social knowledge network of the group, and this network is applied to SMWSNs coverage optimization to transfer information to find the global optimal solution.

### Calculating the fitness value

In solving SMWSNs coverage optimization problems using ACBOA, the fitness value affects the subsequent location updates of individuals in the search space. Butterflies produce fragrances of strength related to their fitness, i.e. when an individual move from one location to another, the individual fitness changes accordingly and the fragrance spreads farther and is perceived by other individuals^39^. In each iteration, all individuals in the search space are moved to a new location and their fitness is calculated, which is the criterion for determining a feasible solution to the coverage problem. The fitness is related to the location of the individuals in the search space, and ACBOA filters the feasible solutions to the coverage problem by the magnitude of the fitness value, which is calculated after the individuals have moved to a new location in each iteration. In the SMWSNs node coverage optimization problem, the fitness value can be calculated by Eq. 9 to obtain the coverage rate of the node.

### Calculating individual butterfly scent perception intensity

In the ACBOA algorithm, the higher the perceptual strength of an individual the greater its ability to attract other individuals, and the perceptual strength of the scent produced by an individual determines the complexity of the subsequent global search phase. Individuals in a butterfly population have their own unique sensory and perceptual abilities, which is a key feature that distinguishes them from other metaheuristics. The intensity of scent perception is generated between individuals and perceived by other individuals as shown in Eq. (12).

12 $$f(x)=c{I}^{a}$$

where $f(x)$ denote the scent intensity function, $c$ denotes the sensory form factor, $I$ denotes the stimulus intensity i.e. the function fitness value, and $a$ denotes the intensity factor, taking values in the range $[0, 1]$.

The convergence speed and individual search method of the ACBOA algorithm are mainly related to the $a$, and $c$ parameters, and they determine the efficiency of the algorithm when applied to the SMWSNs target coverage optimization problem. On the one hand, $a$ of 1 means that the scent emitted by an individual can be sensed anywhere within the monitoring range of the SMWSNs. On the other hand, $a$ of 0 means that the scent emitted by an individual cannot be perceived by other individuals. The sensory morphological coefficient $c$ can theoretically take any value within $[0,\infty )$, and the sensory morphological coefficient $c$ is calculated as shown in Eq. (13).

13 $${c}_{t+1}={c}_{t}+[0.025/({c}_{t}\cdot {T}_{max})]$$

where the initial value of $c$ is 0.01 and ${T}_{max}$ is the maximum number of iterations of the algorithm.

### Global and local position updates

In ACBOA, the behavior of individual butterflies relying on their sense of smell to forage for food symbolizes the process of finding the optimal solution to a SMWSNs coverage optimization problem. When an individual in ACBOA can smell the scent of other individuals, the individual will move in the direction of the strongest scent, which is the global search phase of the algorithm. When an individual cannot perceive the scent from its surroundings it will move randomly and this phase is the local search phase^40^.

During the global search phase, the butterfly moves towards the optimal individual, which is calculated as shown in Eq. (14).

14 $${x}_{i}^{t+1}={x}_{i}^{t}+({r}^{2}\times {g}^{*}-{x}_{i}^{t})\times {f}_{i}$$

where ${x}_{i}^{t}$ is the solution vector of the $i$th individual in the $t$th iteration and ${g}^{*}$ denotes the optimal solution among all solutions of the current iteration. The amount of scent emitted by the $i$th individual is denoted by $f$ and *r* is a random number between $[0, 1]$. The position update in the local search is shown in Eq. (15).

15 $${x}_{i}^{t+1}={x}_{i}^{t}+({r}^{2}\times {x}_{j}^{t}-{x}_{i}^{t})\times {f}_{i}$$

where ${x}_{j}^{t}$ and ${x}_{i}^{t}$ denote the solution vectors of the $j$th and $k$th individuals in the solution space at the *t*th iteration, if ${x}_{j}^{t}$ and ${x}_{i}^{t}$ belong to the same population and $r$ is a random number between $[0, 1]$, denoting a local random wander. The global search and local search process is controlled using a constant switching probability $p\in [\mathrm{0,1}].$

### Cauchy variation

In response to the traditional BOA's tendency to fall into local optima, ACBOA uses Cauchy variation to increase the population diversity, thereby improving the algorithm's global search capability and increasing the search space. The Cauchy distribution function has a small peak at the origin but a long distribution at both ends. Using the Cauchy variation can generate a larger perturbation near the individual butterfly of the current variation, thus making the Cauchy distribution function more extensive, and it is easier to jump out of the local optimum using the Cauchy variation at both ends of the distribution. Traditional BOA incorporates the Cauchy operator to make full use of the effect of the variance at both ends of the Cauchy distribution function to optimally calculate the global optimum individual, making the algorithm better able to reach the global optimum. The standard Cauchy distribution function formula is shown in Eq. (16).

16 $$f(x)=\frac{1}{\pi }(\frac{1}{{x}^{2}+1})$$

After the current optimal solution is obtained, this paper uses Eq. (17) to update the formula for the variation of the current global optimal solution.

17 $${x}_{B}={x}_{best}+{x}_{best}\times Cauchy(\mathrm{0,1})$$

### Adaptive weights

To accelerate the speed of information collection during SMWSNs node monitoring, this paper adds adaptive weights to the ACBOA involved. When the inertia weight is larger, the algorithm has a stronger global search capability, which can increase the population diversity and can search a larger area; when the inertia weight is smaller, the algorithm has a stronger local search capability, which can fine-tune the search around the optimal solution and speed up the convergence speed. In this paper, a new adaptive weight method is proposed, which uses smaller adaptive weights to enhance the butterfly’s local search ability. The adaptive weights are calculated as shown in Eq. (17).

18 $$\omega =1+sin(\frac{\pi \cdot T}{(2\cdot {T}_{max})}+\pi )$$

The above equation $T$ is the current number of iterations and ${T}_{max}$ is the maximum number of iterations. By incorporating an adaptive weighting factor $\omega$, the butterfly individuals are made to have better local search capability, and the local search position update with the addition of adaptive weights is shown in Eq. (19).

19 $${x}_{i}^{t+1}={\omega \cdot x}_{i}^{t}+({r}^{2}\times {x}_{j}^{t}-{x}_{i}^{t})\times {f}_{i}$$

### ACBOA steps

The process of implementing ACBOA is divided into the following eight steps.

Step 1: Initialize the population as well as set the relevant parameters in the algorithm. The population number $pop\_num$, the fitness function $F1$, the maximum number of iterations $MAX\_GEN$, the boundary and the optimization function are changed according to the fitness function.

Step 2: Calculate the scent concentration of each butterfly according to the formula $f(x)=c{I}^{a}$, obtain the fitness value of each individual, and find the current optimal solution.

Step 3: Determine whether the switching probability $p>rand$ or $p<rand$

Step 4: If $p>rand$, perform a global search according to Eq. (1), and perform a Cauchy variation according to Eq. (2) for the global optimal solution generated by the above equation.

Step 5: If $p<rand$, perform a local search according to Eq. (3) and skip to Step 6.

Step 6: Update the individual butterfly and the global optimal solution.

Step 7: If the maximum iteration $T$ is reached output the optimal solution and the fitness value.

Step 8: If the maximum iteration $T$ is not reached then skip to step 2.

**Figure 3:**
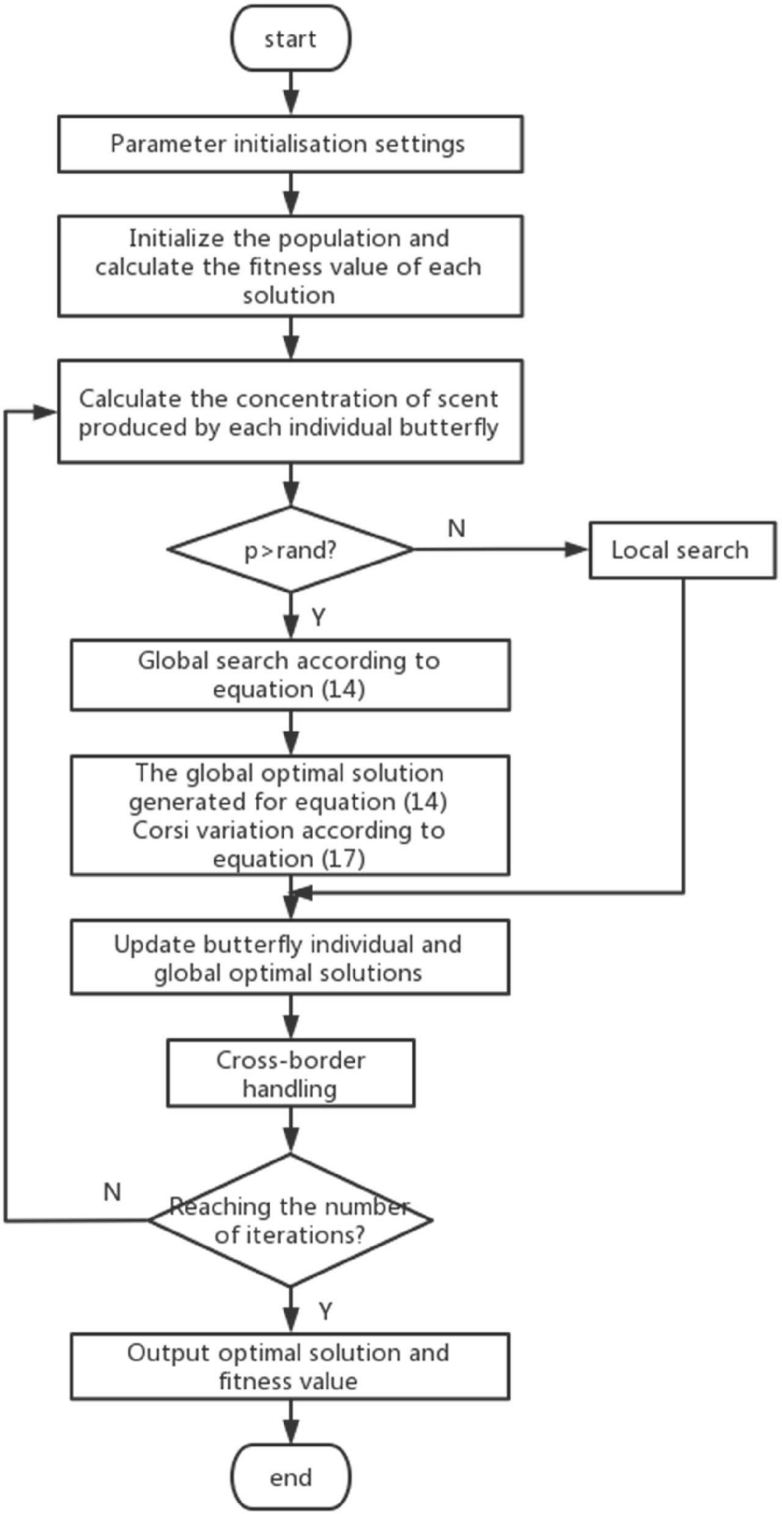
Steps of ACBOA.

The flow chart of the algorithm is shown in Fig. 3.

### ACBOA time complexity analysis

Let the population size is $N$, the number of iterations is $MAX\_GEN$ and the dimension be $D$. According to the operation rules of the time complexity symbol $O$, it can be derived that the time complexity of ACBOA to randomly initialize the population is $O(N\cdot D)$, the time complexity to find the local optimum is $O(N\cdot D)$, the time complexity of the variation of the global location update using the Cauchy distribution function is $O(D)$, the time complexity to introduce the inertia weight factor to update the local position is $O(MaxIter\cdot N\cdot D)$, and the total time complexity of ACBOA is $O(MaxIter\cdot N\cdot D)$. Therefore, the time complexity of ACBOA is equivalent to BOA and does not increase the computational efforts.

## Results and discussion

A series of simulations are conducted to demonstrate the effectiveness of ACBOA in improving the node coverage rate of SMWSNs, which compares ACBOA with BOA, ABC, FOA and PSO. The simulation experiments include comparing coverage rate, node distribution after algorithm optimization, algorithm running time and node energy consumption, which better reflects the practicality of the new algorithm. The hardware environment is uniformly configured with an Intel(R) Core(TM) i5-7200U CPU @ 2.50 GHz computer and Windows 10 operating system, and the simulation software uses MATLAB version 2019a.

To compare the performance of several algorithms in the context of coverage optimization of SMWSNs, uniform common parameters are set to ensure the fairness and objectivity of the experiment. The initial population size $N$ is set to 30 and the number of iterations $MaxIt$ to 500 for all algorithms, under which the proposed new algorithm is compared with other swarm intelligence algorithms. Specifically, in ACBOA, the switching probability $p$ is set to 0.6 to better balance the weight of local and global exploitation, the sensory morphology $c$ is set to 0.01, and the power exponent $a$ is set to 0.1. In BOA, the switching probability is set to 0.8, and the rest of the parameters are set as in ACBOA. In ABC, the number of scout bees $nPop$ is equal to the swarm size, the exploration extremum limit parameter $limit$ is set to 100, and the upper limit of acceleration factor $alim$ is set to 1. In FOA, the search step size $s$ is set to 0.3. In PSO, the velocity range $V$ is $[-\mathrm{2,2}]$, the acceleration factor $c1=c2=2$, the inertia weight $0.4\le wg\le 0.9$, and the discrete granularity $data=1$. The parameters of the above comparison algorithm are set concerning the original classical paper. The specific coverage optimization algorithm parameter settings are shown in Table 1.

**Table 1:** Algorithm parameter setting.

Algorithms	Parameter settings
ACBOA	N = 30, MaxIt = 500, a = 0.1, c = 0.01, p = 0.6
BOA^35^	N = 30, MaxIt = 500, a = 0.1, c = 0.01, p = 0.8
ABC^41^	N = 30, MaxIt = 500, alim = 1, nPop = 30, limit = 100
FOA^42^	N = 30, MaxIt = 500, s = 0.3
PSO^43^	N = 30, MaxIt = 500, Vmax = 2, Vmin = −2, ${c}_{1}={c}_{2}=2$, $0.4\le \mathrm{wg}\le 0.9$, data = 1

The simulation constraints for different monitoring areas, number of nodes, sensing radius and communication radius of sensor nodes are shown in Table 2. Based on the constraints in Table 2, Table 3 shows the network coverage after optimization of the ACBOA, BOA, ABC, FOA, and PSO algorithms. When SMWSNs are applied to actual agricultural irrigation, the communication distance of the nodes is limited due to the environmental occlusion factor, so constraints 1, 2, and 3 are set in the simulation in the communication distance range $8\pm 2$ (m), and constraint 4 is set in the simulation in the communication distance range $4\pm 1$(m). As long as the communication distance in the simulation result is within the limit, the communication is considered valid. To investigate how the nodes react in case of failure, a uniform failure rate of 5% is set when the nodes are deployed.

**Table 2:** Four types of restrictions.

	Monitoring area	Number of nodes	Sensing radius	Communication radius
Restriction 1	50 × 50	53	5	$10\pm 2$
Restriction 2	50 × 50	42	5	$10\pm 2$
Restriction 3	50 × 50	32	5	$10\pm 2$
Restriction 4	20 × 20	26	2.5	$5\pm 1$

**Table 3:** Coverage efficiency with different monitoring area size, number of nodes, sensing radius and communication radius.

	Restriction 1 (%)	Restriction 2 (%)	Restriction 3 (%)	Restriction 4 (%)
ACBOA	99.46	95.67	83.47	91.16
BOA	90.37	83.92	71.71	80.66
ABC	85.68	77.65	66.47	78.66
FOA	96.89	91.89	79.55	85.03
PSO	88.35	88.50	67.28	87.08

**Figure 4:**
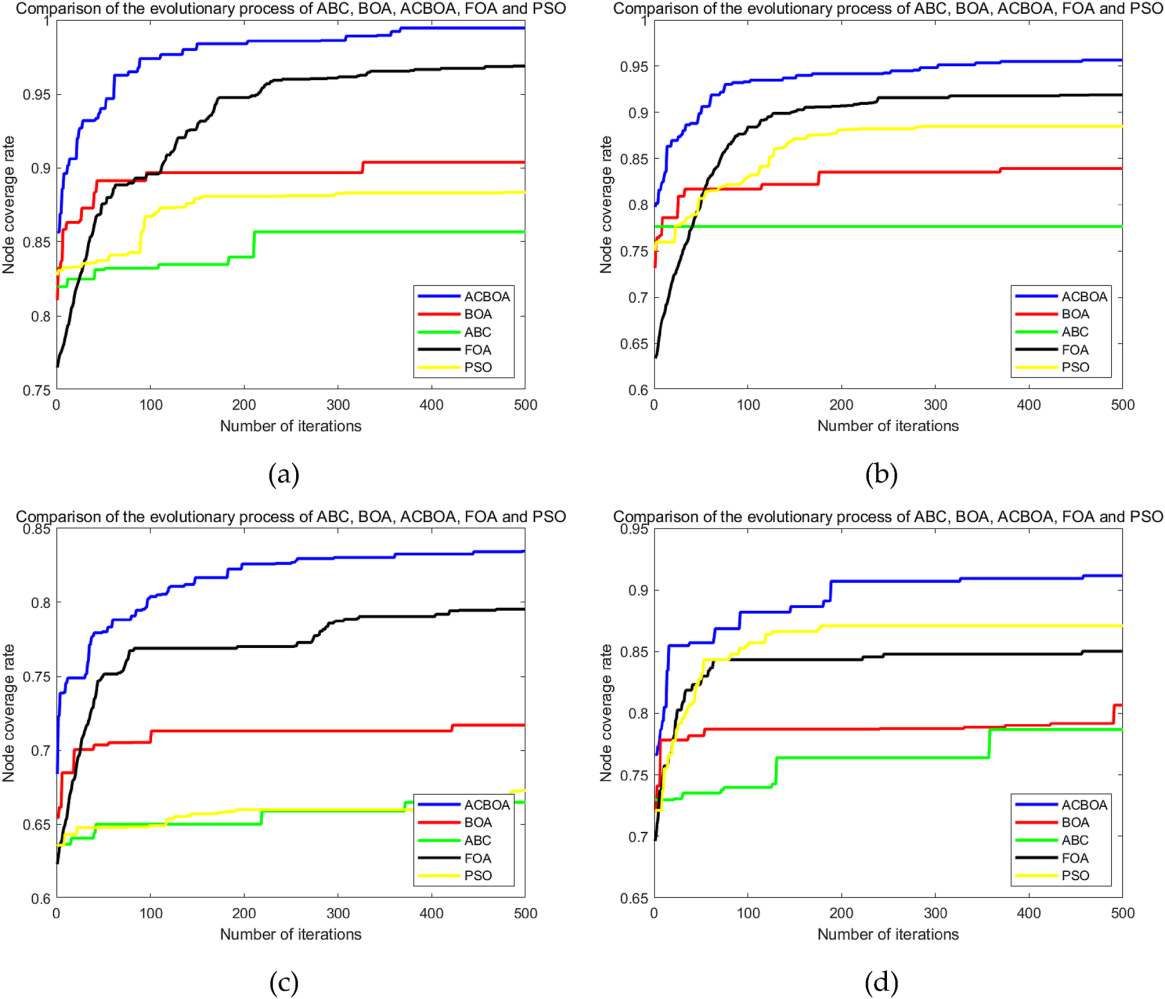
Iterative curves of coverage optimization under different conditions (**a**) Restriction 1, (**b**) Restriction 2, (**c**) Restriction 3, (**d**) Restriction 4.

Figure 4a–d show the simulation results of ACBOA, BOA, ABC, FOA and PSO for node coverage optimization in SMWSNs under four different constraints. From Fig. 4a–d, it is clear that the optimized node coverage of ACBOA has the highest node coverage rate and is applied to the best monitoring quality in SMWSNs. Figure 4a shows that the node coverage rate after ACBOA optimization are better than BOA, ABC, FOA and PSO. The coverage rates of BOA, ABC, FOA and PSO after deploying sensor nodes are 90.37%, 85.68%, 96.89% and 88.35%, respectively. When the improved ACBOA algorithm is utilized in the coverage optimization of SMWSNs, the sensor nodes are evenly distributed in the monitoring area and the coverage rate reaches 99.46%, which is 9.09%, 13.78%, 2.57% and 11.11% higher than the coverage rates of BOA, ABC, FOA and PSO algorithms, respectively. In addition, the coverage rate of BOA, ABC, FOA and PSO algorithms reached 90.21%, 83.37%, 90.11% and 86.93%, respectively, at 100 iterations in terms of the optimization effect, the coverage rate of nodes after ACBOA optimization has reached 97.25%, which is 7.04%, 13.88% and 7.14% higher than the coverage rate of BOA, ABC, FOA and PSO algorithms. 13.88%, 7.14% and 10.32% compared to BOA, ABC, FOA and PSO algorithms. This comparison result indicates that the improved strategy can effectively improve the algorithm's merit-seeking ability and achieve better coverage with a smaller number of iterations.

Figure 4b reduces the number of nodes by 11 compared to the condition in Fig. 4a. The node coverage after ACBOA optimization in Fig. 4b is 95.67%, which is 5.3%, 9.99% and 7.32% higher compared to the results after BOA, ABC and PSO coverage optimization in Fig. 4a, respectively. The simulation results of both figures show that with the reduced number of nodes deployed in the coverage optimization of SMWSNs, ACBOA can still achieve better node coverage and effectively reduce the network deployment cost.

**Figure 5:**
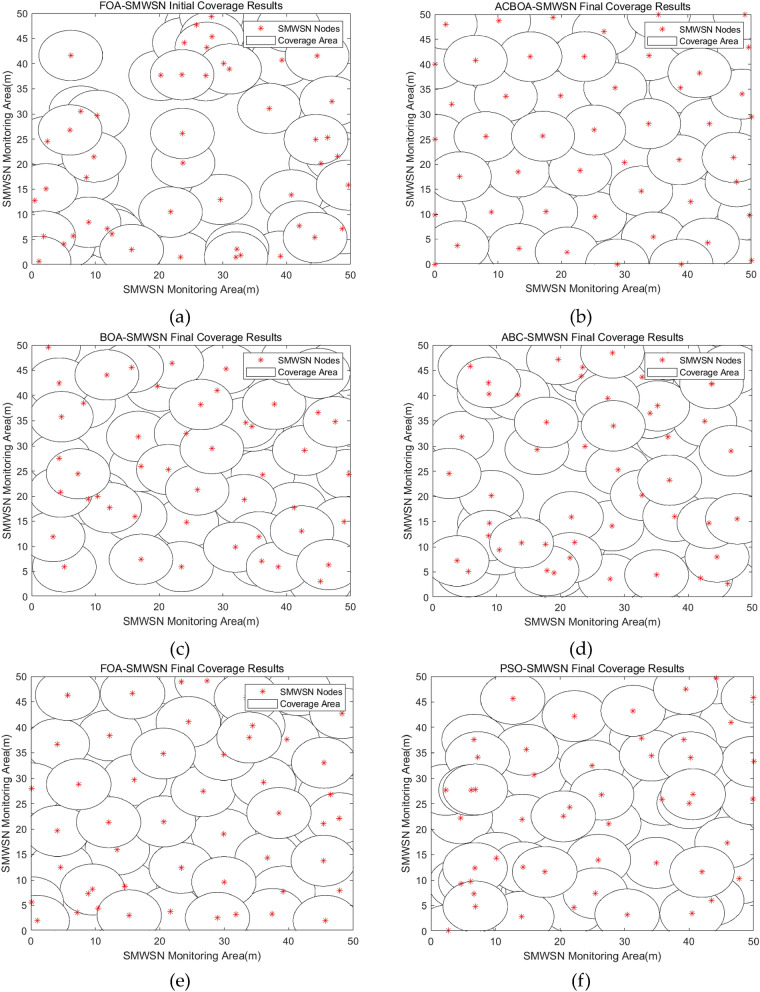
Node deployment when monitoring area 50 m × 50 m, number of nodes 53, sensing radius 5 m, communication radius $10\pm 2$ m (**a**) Random deployment, (**b**) ACBOA optimization, (**c**) BOA optimization, (**d**) ABC optimization, (**e**) FOA optimization, (**f**) PSO optimization.

**Figure 6:**
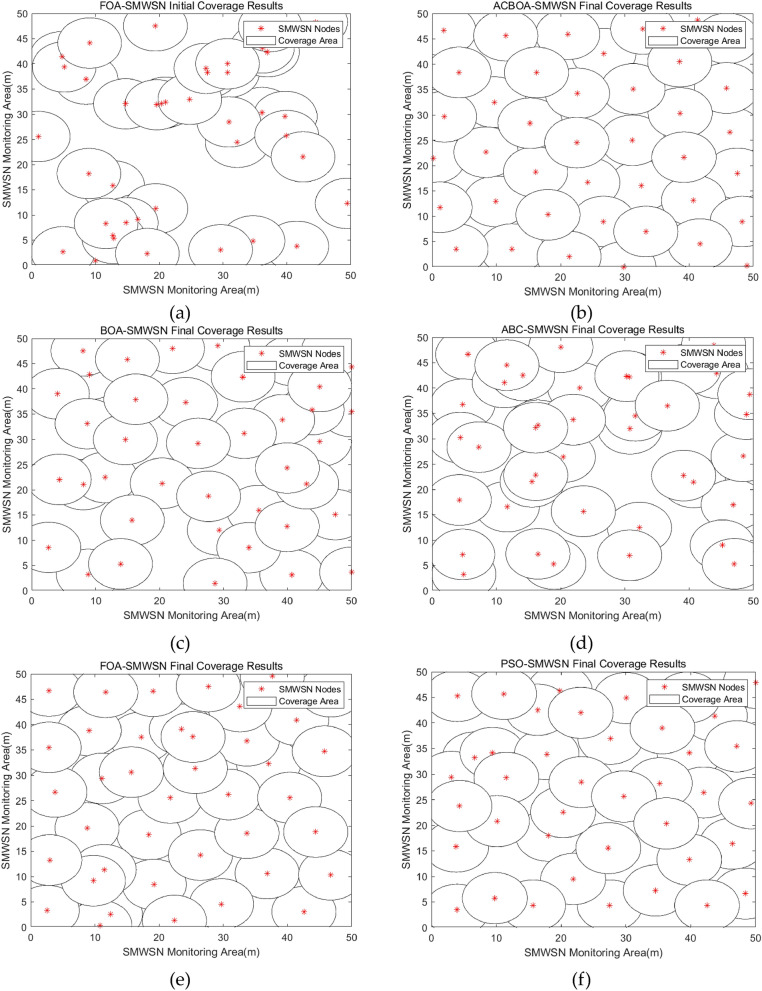
Node deployment when monitoring area 50 m × 50 m, number of nodes 42, sensing radius 5 m, communication radius $10\pm 2$ m (**a**) Random deployment, (**b**) ACBOA optimization, (**c**) BOA optimization, (**d**) ABC optimization, (**e**) FOA optimization, (**f**) PSO optimization.

Figures 5 and 6 compare the results of optimized deployment of ACBOA, BOA, ABC, FOA, and PSO for SMWSNs nodes under constraints 1 and 2. To facilitate the comparison of the node distribution before and after the optimized deployment, the deployment results of FOA nodes with the lowest initial coverage rate of all algorithms are compared with the deployment results of the remaining algorithms after optimization. According to Figs. 5 and 6, it is known that the influence of the number of nodes on the coverage is explored under the same constraints of monitoring area, node sensing radius and communication radius. From the simulation results, it can be concluded that the more the number of nodes in the appropriate range the higher the coverage rate, the less coverage redundancy and coverage hole phenomenon in the monitoring area. In Fig. 5a, the nodes are randomly thrown in the monitoring area, and the results show that the randomly deployed nodes are extremely unevenly distributed and there are a lot of coverage redundancy and coverage holes. In Fig. 5c–f, the optimized deployment results using BOA, ABC, FOA and PSO show a more uniform distribution of nodes, but there are still more redundant nodes and coverage blanks, the coverage of nodes optimized by both algorithms still needs to be improved. In Fig. 5b, the results of applying ACBOA algorithm to node coverage optimization show that the nodes are more evenly distributed and the coverage voids are greatly reduced, and the area can be approximated to achieve complete coverage with the best coverage effect.

Furthermore, the coverage rate after ACBOA optimization under constraint 1 is higher than the coverage rates of BOA, ABC, FOA, and PSO under constraint 2, as well as dramatically reducing the coverage blindness of the deployment results. This finding demonstrates that the coverage rate can be effectively improved with a greatly reduced number of deployed nodes, proving the effectiveness of ACBOA in improving the node coverage rate. A comprehensive comparison of Figs. 5 and 6 shows that the optimized nodes of ACBOA have the most uniform and reasonable distribution and the highest utilization of nodes.

The results of the simulation experiments in Figs. 5 and 6 show that the nodes that fail in the actual simulation go dormant, so the actual number of nodes involved in the deployment optimization in Figs. 5 and 6 are 53 and 42, respectively.

**Figure 7:**
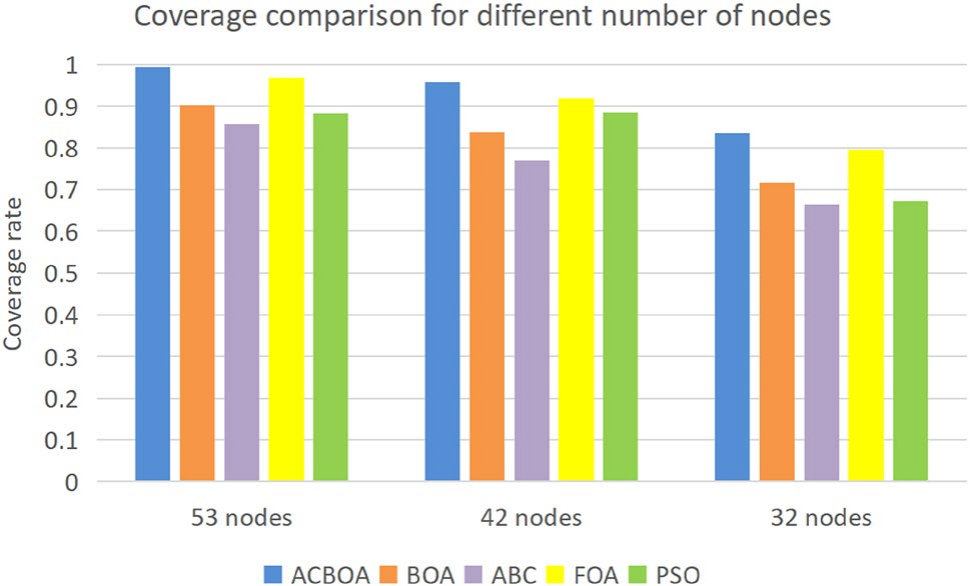
Coverage comparison.

Figure 7 shows the change in coverage rate of the ACBOA, BOA, ABC, FOA and PSO optimization algorithms as the number of sensor nodes in the SMWSNs decreases. At a node count of 53, the coverage rate of the five optimization algorithms is 99.46%, 90.37%, 85.68%, 96.89% and 88.35% respectively. With a node count of 42, the coverage rates of the five optimization algorithms were 95.67%, 83.92%, 77.65%, 91.89% and 88.50% respectively. With a node count of 32, the coverage rate of the five optimization algorithms was 83.47%, 71.71%, 66.47%, 79.55% and 67.28% respectively. This simulation constraint differs only in the number of nodes, with the coverage rate still reaching a high value for the number of working nodes of 53, compared to the coverage rates of BOA, ABC and PSO for the premise of 42 working nodes. The difference in coverage rate with FOA is only within a small range. The results show that compared to the four compared algorithms, ACBOA significantly improves the coverage rate of SMWSNs nodes with a significant reduction in the number of deployed nodes, which not only saves the configuration cost of the network but also reduces the energy consumption.

**Figure 8:**
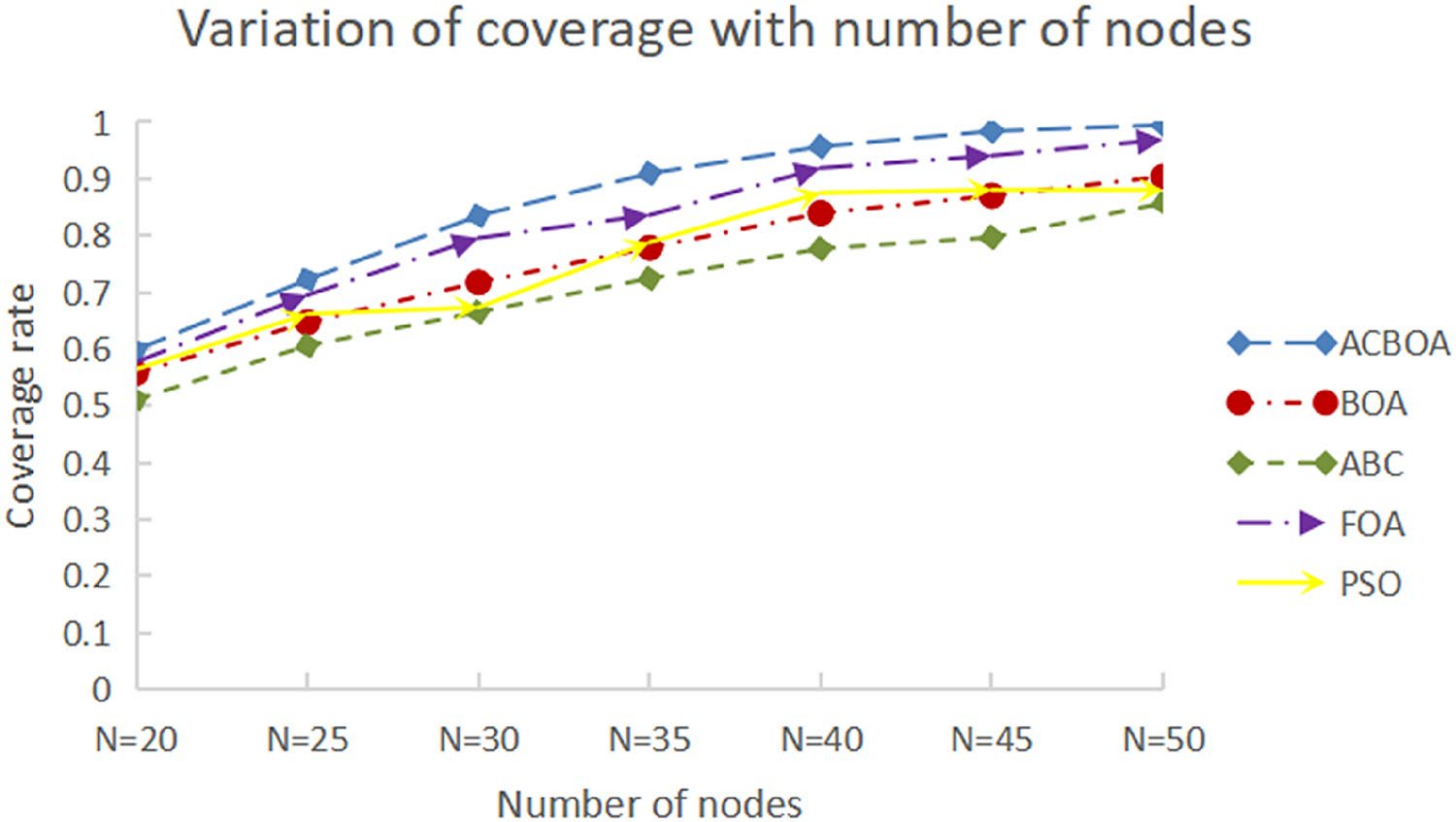
Variation of coverage with number of nodes.

Figure 8 explores the impact on coverage for five node deployment strategies, ACBOA, BOA, ABC, FOA and PSO, when changing only the number of nodes for 500 iterations. It can be seen from the figure that the node coverage tends to increase with the number of nodes under the restriction 1. The network coverage rate of ACBOA is higher than that of BOA, ABC, FOA and PSO algorithms when the number of nodes is the same. Such experimental results indicate that ACBOA can better adapt to the changes in the number of sensor nodes under the same parameter conditions, and it has a wider coverage optimization application range. As the number of sensor nodes increases, the coverage of sensor nodes optimized with ACBOA increases and the network resource consumption decreases. In addition, the increased coverage indicates that the monitoring quality of the network is improved and the collected data are more accurate, which makes ACBOA suitable for coverage optimization of SMWSNs.

**Figure 9:**
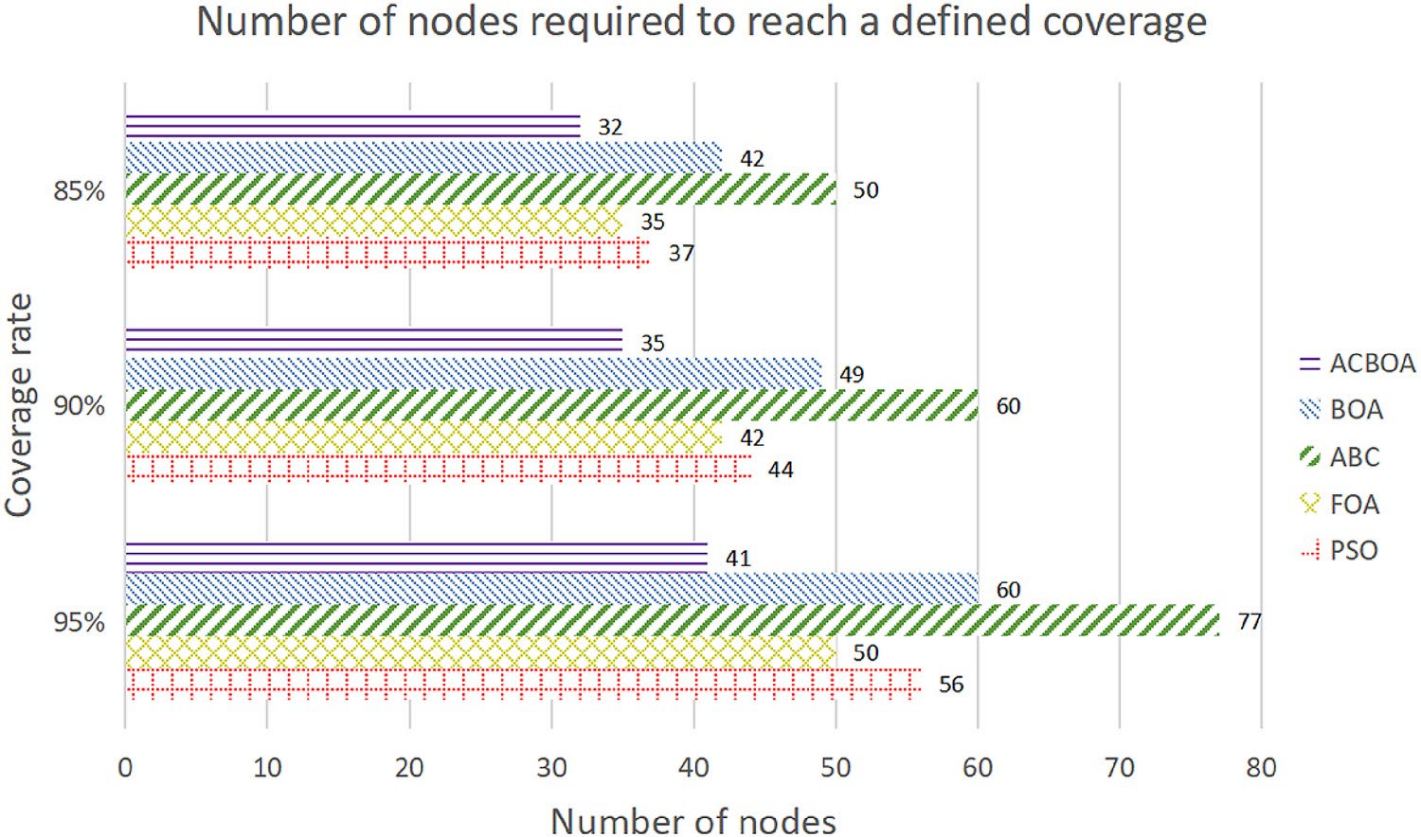
Number of nodes required to reach a defined coverage.

Figure 9 represents the calculation of the number of nodes that need to be deployed to reach the determined coverage after optimizing node deployment with the five algorithms ACBOA, BOA, ABC, FOA and PSO under constraint 1. As shown in the figure, only 41 nodes need to be deployed for the network optimized with ACBOA when the coverage reaches 95%, while 60, 77, 50, and 56 nodes need to be deployed with BOA, ABC, FOA, and PSO, respectively. Too many nodes deployed by the four compared algorithms are prone to coverage redundancy, which affects the accuracy of data transmission. The energy consumed by all nodes in the network for 1 h of operation is calculated according to Fig. 9, as shown in Table 4. It can be seen that the higher the number of sensor nodes the higher the energy consumption at the same communication radius. The nodes exhibit the highest energy consumption after the optimal deployment of ABC algorithm under the three conditions, and the nodes exhibit the least energy consumption after the optimal deployment of ACBOA. Proposed ACBOA algorithm in this paper achieves the purpose of reducing the energy consumption of the network.

**Table 4:** Average energy consumed by all nodes working for one hour.

	85% (J)	90% (J)	95% (J)
ACBOA	0.096	0.105	0.123
BOA	0.126	0.147	0.18
ABC	0.15	0.18	0.231
FOA	0.105	0.126	0.15
PSO	0.111	0.132	0.168

The total running time of the algorithm iterative optimization search with constraint 1 is shown in Table 5. The running time of ACBOA is smaller than BOA, ABC, FOA and PSO algorithms, which indicates that ACBOA has the shortest iterative search time and the highest running efficiency. The adaptive factor introduced by ACBOA can quickly escape from the loop and tend to the optimal solution space when it falls into local optimum, which greatly accelerates the convergence efficiency and running efficiency of the algorithm.

**Table 5:** Algorithm running time.

Algorithms	Running time (s)
ACBOA	21.524244
BOA	44.716852
ABC	148.989030
FOA	32.716852
PSO	92.148674

## Conclusions

To optimize the node coverage of SMWSNs, an adaptive Cauchy butterfly optimization algorithm (ACBOA) is proposed in this paper, with an important innovation that adaptive operators and Cauchy variants for improving the global and local search ability, respectively, are presented to effectively improve the node coverage. In addition, a new method of constructing an objective function optimization model using the effective coverage of nodes as an optimization factor was devised, and the node coverage problem was transformed into an objective function optimization problem was proposed. Subsequently, ACBOA is compared with BOA, ABC, FOA and PSO algorithms to show the effectiveness of the five algorithms for coverage optimization of SMWSNs under different constraints. Besides, the energy consumption and running time of the five algorithms are also compared in this paper. The simulation results show that the ACBOA based coverage optimization outperforms the other algorithms in terms of optimization capability and saving network resources. In addition, ACBOA is suitable for large-scale sensor networks, the application in coverage optimization of SMWSNs effectively improves node coverage while reducing the number of nodes deployed, which provides a possibility for further development in precision agriculture.

Future research should consider more complex SMWSNs including but not limited to exploring the impact of different depths of collected soil moisture data by solving the same optimization model in simulation using an optimization solver, e.g., GLPK or CEPLEX. in addition, this paper is only based on MATLAB software for simulation, and subsequent testing of the sensor optimization deployment in a real farm environment can be conducted to explore the execution time of different algorithms on the sensors.

## Data Availability

The datasets generated during this current study are not publicly available due to privacy, but are available from the corresponding author on reasonable request.
